# Novel Academic Tabletop 2022 (NAT22): A Dynamic Dice-Based Emergency Medicine Education Tool

**DOI:** 10.7759/cureus.49498

**Published:** 2023-11-27

**Authors:** Adam Heilmann, Jessica Pelletier, Collyn Murray, Alexander Croft

**Affiliations:** 1 Emergency Medicine, Washington University School of Medicine, St. Louis Children's Hospital, St. Louis, USA; 2 Emergency Medicine, University of North Carolina at Chapel Hill School of Medicine, Chapel Hill, USA

**Keywords:** simulation medicine, gamification technique, dungeons and dragons, teaching in emergency medicine, simulation in medical education, tabletop roleplaying game

## Abstract

Gamification is an effective teaching tool that improves engagement and knowledge retention. Tabletop role-playing games are dynamic games that use random chance and foster player/leader partnership. To date, there are no teaching tools that mimic dynamic or unpredictable patient presentations. This style of game may work well as a tool for medical education in a simulation-based modality.

In this report, we document the rules, materials, and training required to reproduce a hybrid game created to combine facets of simulation and tabletop role-playing games (TRPGs) to create a dynamic medical education tool.

After testing the game for flaws and fluidity of gameplay, we plan to collect data evaluating emergency medicine residents' enjoyability and knowledge retention. In this article, we describe a novel TRPG simulation hybrid game that we hypothesize will improve learner enjoyability/engagement and have similar educational benefits to standard medical education.

## Introduction

Your players are trapped in a cave surrounded by goblins ready to attack. The group’s fighter tries to move the rubble, blocking the exit. “Roll a strength check,” you ask as the player rolls the 20-sided dice and adds their strength bonus. “Eight” is not enough to perform the task; you describe the fighter mustering all their strength to no avail. With no way out, the party must prepare to fight.

Roll for initiative

Since the 1970s, tabletop role-playing games (TRPGs) such as Dungeons and Dragons® have entertained adults and children alike using imaginative dice-based scenarios as described above. Interpersonal communication, folklore and mythology, and cultural competencies have all been modeled using the TRPG-based systems [1]. TRPGs are role-playing games that do not use a board but rather dice and improvisation to generate storytelling. Typically, characters are created and scenarios are proposed, so characters can improvise solutions or interactions using combined storytelling. These games utilize random chance and probability from dice rolls to determine player outcomes in different situations. The dynamic nature of TRPGs creates a unique gaming environment in which neither the player nor the session leader knows what will happen next. TRPGs and other role-playing games have been recognized to have the potential to be a strong teaching methodology in the realms of psychology and education [2]. Emergency medicine education already utilizes role play as an effective teaching tool for patient scenarios and difficult discussions. TRPGs may be a viable medical teaching tool as well.

Graduate medical education has started to study and incorporate democratic teaching modalities, where educators partner with learners and are seen as facilitators rather than instructors. This learner-facilitator partnership generates an engagement that is less seen in classical didactic learning. Brown et al. described TRPGs as an effective learning tool, comparing many similarities between the framework of Dungeons and Dragons® with principles of graduate medical education [2]. There have been multiple studies evaluating the use of gamification in disseminating educational content [3-6]. However, to date, there has not been an adaptation of gamification with simulation, a teaching modality well suited for this adaptation. As TRPGs have similar mechanics to simulation, there is potential for incorporating gamification into medical simulation [7].

Emergency medicine is a dynamic field requiring frequent readjustment, mastery of task prioritization, and frequent reorientation to patient presentations. It can be difficult to reliably reproduce scenarios that challenge these skills in a didactic or active learning setting [8,9]. There is a paucity of information on dynamic and tabletop teaching modalities that specifically challenge a learner’s ability to task-prioritize and task-switch. This educational gap was the inspiration for the creation of a new gamified teaching tool. By fusing gamification and simulation in a way that is dynamic and novel, we may be able to challenge learners in a new way that is engaging and educational for difficult patient presentations. With this in mind, we have developed a tool combining TRPGs and simulation called the Novel Academic Tabletop of 2022 (NAT22).

In the NAT22 model, learning objectives are set, but there is no predefined pathway. Learners may have to repeatedly reassess patients, be interrupted by a crashing patient, or move to second- or third-line therapies for patients who do not respond to initial management. Because of the case-to-case variability, a single case can have significant flexibility in its targeted learners while still meeting learning objectives, specifically while meeting different learners near their specific zone of proximal development [10,11]. We hypothesize that this model will be an engaging and effective tool for replicating dynamic patient encounters. This model may also be adapted to simulate team-based interactions to broaden its impact on interprofessional development.

## Technical report

Setting

This study was performed virtually and in person at Washington University in St. Louis School of Medicine with emergency medicine residents in postgraduate years (PGY) one and two. The sessions did not require a significant amount of space and could be performed at a standard-sized desk with minimal materials. Virtually, we utilized a standard Zoom® video-conferencing platform. Each session lasted between 35 and 50 minutes. There is variation in timing due to the variation in gameplay.

Materials/personnel

All materials can be provided virtually or in person. Each session consists of one learner and one proctor. Participants require a computer with a webcam and microphone if participating virtually. Both live and virtual sessions require paper and a pen as well as either two six-sided dice or dice-rolling software on an electronic device. Finally, each participant will need a copy of the rules and the stat block based on their PGY that dictates which bonuses they have for their dice rolls (Table 1).

**Table 1:** Material required for in-person and virtual gameplay

Materials required	
Virtual	In person
Computer with webcam and internet connectivity	Dedicated space for two people
Video-conferencing software	Pen and paper
Presentation with patient media and laboratory values	Printouts of patient images and laboratory values
Dice-rolling software	Two six-sided dice for the learner and proctor

Training the trainer

Three sessions were set up to train proctors before running the simulation and collecting data. Although the case is meant to be dynamic, the proctor must be able to understand the general rules of the game and improvise scenarios that fit dice rolls. Each first-time proctor had an initial session where they played as a learner. The gameplay was discussed with each instructor, and they then ran a session with the experienced proctor serving as the learner. All feedback was reviewed, and a final session was held with a third-party physician who was not involved with the NAT22 team. For those implementing our protocol, more practice sessions can be added as needed if rules are unclear or if there are other questions.

Activity description

The game starts with a pre-brief. The pre-brief details the rules of the game and explains that because of the innate randomness of the exercise, there is a chance that the patient will get worse or even code despite proper care. Participants are instructed that if at any point they feel uncomfortable, they can pause or stop the simulation. The pre-brief is intended to establish psychological safety, specifically with facilitated patients who may get more unstable and perish. An initial, brief description of a patient is then given including their age, sex, and chief complaint. The game is turn-based with the learner taking their turn first. Learners have two actions they can perform on their turn. These actions include any patient care task. Examples include taking a history, checking vital signs, performing a physical exam, administering medication, or obtaining labs/imaging. Actions are placed in one of three categories: Medical Knowledge (MedKnow), Procedure (Proc), or Quick (Qui). A full list of actions and categories is listed in Table 2. Following the player's two actions is the patient's turn. During this phase, the proctor rolls the dice for the patient to determine if the patient responds to therapy, remains unchanged, or decompensates. The turns continue until the player declares they want to take the “disposition” action, either admitting, discharging, or transferring the patient out of the emergency department.

**Table 2:** An example list of actions and their categories EMS: Emergency Medical Services.

Actions		
Quick (QUI)	Procedure (PROC)	Medical Knowledge (MEDKNO)
History	Therapy	Vitals
Call consult	Procedures	Physical exam
Talk to family/EMS	Disposition	Labs/Imaging

Players turn

When the player states the action that they wish to take during their turn, they roll two six-sided dice to determine the outcome of the action. Three categories of rolls exist: low tier (roll of 2-5), middle tier (roll of 6-9), and high tier (roll of 10-12). The higher the roll, the better the outcome. For example, a low-tier roll to check vital signs may result in only one vital sign given, whereas a high-tier roll gives all vitals. For a full list of roll tiers and examples, refer to Table 3. After the player rolls, they add any applicable bonuses. A roll under the “Quick” category would add a player’s “Quick” bonus listed on their bonus sheet.

**Table 3:** A list of potential dice results and the corresponding results

Dice results and subsequent in-game actions	
2-5	Failure: severe compilation or little to no information given.
6-9	Partial success: complete action with some information or minimal complications.
10-12	Full success: complete action with all required data and/or no complications.

Each player receives a stat sheet that lists some bonuses they may add to specific rolls. The bonuses range from +1 to +3. Each category (Quick, Medical Knowledge, and Procedure) has its own bonus, and any roll within each category gets the corresponding bonus applied (Table 4). They are added after the player rolls the dice to increase the likelihood of a high-tier roll. These bonuses decrease as the level of the learner increases, improving the replayability of a single case for learners at different stages. So, a PGY4 may have no bonuses, making the same case more likely to have lower rolls and thus more difficult.

**Table 4:** Bonuses applied to rolls for specific actions. Each level of learner (indicated by PGY) has different bonuses. This allows one case to be used for different learners while keeping an appropriate level of difficulty. PGY: Postgraduate year; Resus: Resuscitation.

Quick	Procedure	Medical Knowledge
PGY1		
3	2	2
Resus points	1	
PGY2		
2	1	2
Resus points	1	
PGY3		
1	1	1
Resus points	1	
PGY4		
0	0	0
Resus points	0	

Patients (facilitators) turn

**Figure 1:**
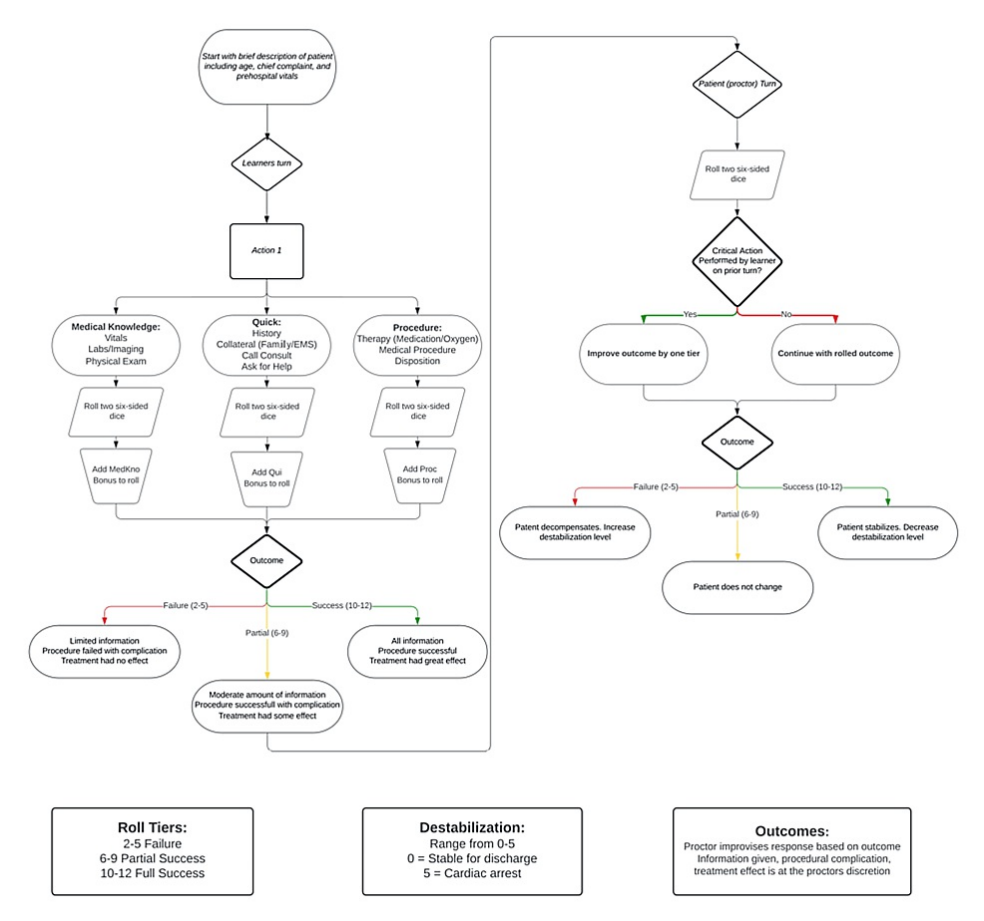
Gameplay flow sheet generated by authors outlining the order of operations for gameplay. Modifiers are included and definitions for rolls, destabilization points, and proctor actions are listed.

After the player’s turn, the facilitator takes a turn as the “patient.” The patient initially presents with a destabilization level on a scale from 0 to 5 that is revealed to the player at the beginning of the play, 0 is completely stable, and 5 is pulseless. The “patient” will roll dice to determine if they destabilize or stabilize. To keep gameplay fair, when a player performs a critical action (specifically written for each case), the patient’s dice roll will be bumped to the next highest tier to prevent unnecessary destabilization. It is up to the facilitator to narratively describe to the player clinically relevant reasons why the patient would destabilize on a poor roll and how they would stabilize on a successful role. Refer to Figure 1 for the gameplay flowsheet.

Resuscitation points

Players can obtain resuscitation points for completing critical actions in a timely manner or catching easy-to-miss exam findings. These points are awarded at the facilitator’s discretion based on the learner’s performance. Resuscitation points can be used to decrease the destabilization level of the patient by one. Resuscitation points can be played anytime even if it is not the player's turn.

Special actions

A player can take a few actions that have more detailed rules. For example, the procedure action is more involved than simply asking to take vital signs. If the player wishes to perform a procedure, they must first describe the materials needed and how to perform it. With a low-tier roll, the procedure is not completed due to a complication (invented by the proctor), and the player must address this complication in the following action. A medium-tier roll allows completion of the procedure with complications, and a high-tier roll is completion with no complications. If a complication arises, the proctor must identify which elements of the setup have been missed or forgotten by the learner and base the complication on that. For example, if the procedure is intubation, a potential complication could include vomiting if the learner forgot to verbalize the necessity of preparing suction prior to the roll.

Debriefing

All learners are presented with a standardized debrief for the case. Debriefing, done using debriefing with good judgment [12], is completed after the sessions according to the learning objectives of the case (Table 5).

**Table 5:** Learning objectives of the case This table debriefs the learning points with specific questions and topics to review. Similar to other teaching modalities, this study allows a standardized post-session review to ensure that learning points are met. CXR: Chest X-ray; PPV: Positive pressure ventilation; HF: Heart failure; ACS: Acute coronary syndrome; PE: Pulmonary embolism; CHF: Congestive heart failure.

Objectives	
Educational goal	Promptly identify the signs and symptoms of heart failure exacerbation. Manage the airway of a patient with flash pulmonary edema. Understand pharmacologic therapies for severe CHF in the emergency setting.
Medical objectives	Describe procedures such as intubation and initiation of bi-level ventilation. Understand dosages of specific medication for intubation, blood pressure control, and afterload reduction. Identify other triggers for HF exacerbation (ACS and PE).
Management objectives	Prioritize the treatment of volume overload. Maintain airway with frequent reassessment. Identify key laboratory markers and imaging studies to aid in diagnosis.
Questions to review	
What is the diagnosis and how did you come to it?	
Describe your airway management. Would you have done anything differently?	
What was your first-line medical treatment for the patient in respiratory distress from volume overload?	
What are some interventions if a patient with sympathetic crashing adrenergic pulmonary edema becomes hypotensive?	
Key moments/critical actions	
CXR/ultrasound for volume assessment, initiate diuresis, PPV for ventilatory support, and address chest pain.	

## Discussion

As stated in the early critical reasoning literature, “Uncertainty is an unavoidable aspect of the human condition. Many significant choices must be based on beliefs about the likelihood of such uncertain events as … the outcome of a medical operation…” [13]. By creating an environment that allows learners to explore uncertainty via diagnostics or treatments, we can mimic this phenomenon in a controlled setting, thereby challenging a learner's critical thought process. Additionally, despite an individual's medical knowledge, their application of this information through judgments and interpretations of patient presentations, laboratory values, or other data, may differ widely. It is important to assess or challenge one’s ability to interpret uncertainty to reveal biases and heuristics that may influence their critical thinking skills [14].

“Critical thinking,” however, is a broad term. There is debate over what exactly contributes toward thinking critically and whether it is a knowledge or skill-based action. Edwards detailed a two-phase framework: First, a knowledge-based domain is described as experiences one learns from, and second, a skill-based domain is described as the ability to demonstrate a flexible mindset in the face of complex problems [15]. Regardless, the ability to apply knowledge to complex situations is a key component of medical education, which significantly affects patient outcomes [16]. Challenging learners in this space allows them to practice utilizing the aforementioned cognitive processes in a clinical environment with simulated consequences to their actions.

The NAT22 format attempts to directly challenge both domains of critical thinking. Participants will have to stretch their medical knowledge to apply concepts in unique/complex patient presentations. By limiting learners to only a few actions before a patient decompensates and allowing for multiple clinical courses, NAT22 necessitates flexible thinking and dissuades generalizable pattern recognition. Throughout this heuristic process, learners may gain insight into their own biases that influence decisions. The NAT22 educational tool was designed to stress a learner's critical thinking ability to an extent that traditional teaching cannot.

We continually engage learners by incorporating dynamic patient encounters to provide challenging educational experiences. While there have been many attempts at gamification of core content including escape rooms, board games, and even longitudinal national competitions [17-19], only little data evaluating stochastic learning, specifically in emergency medicine, is available [20]. Many arenas of healthcare education advertise the implementation of critical thinking or reasoning mostly through case-based or problem-based learning. These tools are effective in their own respect but may not fully elicit the mental fluidity and flexibility required to manage complex patients.

Although this innovation is in its infancy, NAT22 has the potential to collect and analyze data in a way that standard teaching modalities cannot. In its turn-based system, decisions can be tracked and corrected appropriately for high-/low-tier rolls. Tracking turns or timing may be beneficial in evaluating efficiency, task prioritization, or other advanced practice patterns. The ability to modify plans in a time-limited scenario is another facet of medicine that is difficult to replicate and evaluate. NAT22 offers a low-cost, high-fidelity alternative that may provide these benefits. Alternatively, data can be evaluated to teach or provide feedback on residents’ clinical reasoning. Manipulating the cases to have numerous potential diagnoses may give an insight into learners’ clinical practice. Although these components have not yet been tested, this style of gamification opens the door to a myriad of possible new learning domains.

Initial pilot testing was successful in terms of gameplay and mechanics. This technical report is a precursor to data collection meant to disseminate NAT22 in a “plug and play” fashion. Our version began with one case that was performed with first- and second-year residents. This was intended to limit variability and test the game prior to making multiple cases of varying difficulties. Each case was recorded and reviewed to identify inconsistencies to be improved for future versions.

A few limitations exist within NAT22. Beta testing before data collection will help us to identify areas of improvement before going live with participation. There are a significant number of rules in NAT22, which makes the game flow well and remain balanced, but this could be difficult for learners to grasp promptly. Additionally, facilitator creativity and improvisation are required. For example, if a player performs all actions appropriately but the patient decompensates from random chance, not all preceptors may explain the decompensation the same way. This may cause the learner to go down two different treatment pathways. Although this is different from traditional simulation, it is an intentional benefit of the game generating unique scenarios, requiring critical thinking and making it repeatable with numerous outcomes/scenarios. However, this also can generate variability in the timing, requiring additional logistical thought when planning educational activity.

Future directions include data collection using a mixed methods approach analyzing both qualitative and quantitative measures. Quantitative measures will include a multiple-choice questionnaire and Likert scale data. The multiple-choice questions will be completed both pre- and post-simulation to gauge medical knowledge. The Likert scale will be a five-point scale that will be disseminated post-intervention and designed to assess feasibility, satisfaction, and difficulty. Improvement will be evaluated on a Kirkpatrick 1 and 2 level, assessing reaction and learning. Qualitative data will be collected and coded from the standardized debrief session following each session similar to a conducted interview. All data are to be deidentified and collected using secure Qualtrics software (Qualtrics, Seattle, Washington).

## Conclusions

This format of gameplay is novel and creates an engaging learning environment. The primary goal of creating this tool is to train learners dynamically while promoting a flexible mindset. With the intent of maintaining an equal level of core medical content teaching, NAT22 adds the ability to practice critical reasoning in a simulated environment. Additionally, the low cost and ease of trainer training make this a widely implementable learning activity. We hypothesize that this innovation will increase learner engagement, while improving complex patient care skills, and to date, there is not a specific gamified protocol to practice these skills. This game may aid in direct clinical practice, preparing learners for critically ill patient encounters, and provide a similar level of teaching when compared to traditional modalities such as simulation and oral board cases. Full data will be collected, and analysis will include multiple-choice pre- and post-tests, feasibility data, satisfaction, and qualitative interview data. Combined quantitative and qualitative data analysis will be performed to identify the potential benefits in the reaction, learning, and behavior domains.
